# Biogenic Metal Nanoparticles from Indian Flora as Programmable Bio-Interfaces: From Phytochemical Coronas to Precision Nanomedicine

**DOI:** 10.3390/ijms27135837

**Published:** 2026-06-28

**Authors:** Sharad Shriram Tat, Kailas D. Datkhile, Jayant R. Pawar, Amar R. Mohite, Tanisha Sharma

**Affiliations:** 1 Department of Pharmacology, Krishna Institute of Medical Sciences, Krishna Vishwa Vidyapeeth (Deemed to be University), Karad 415 539, Maharashtra, India; 2 Krishna Institute of Science and Technology, Krishna Vishwa Vidyapeeth (Deemed to be University), Karad 415 539, Maharashtra, India; jayantpawar26@gmail.com (J.R.P.); armohitept@gmail.com (A.R.M.); 3 Department of Biochemistry and Structural Biology, Greehey Children’s Cancer Research Institute, University of Texas Health Science Center at San Antonio, San Antonio, TX 78229, USA; sharmat@uthscsa.edu

**Keywords:** phytochemical corona, nanomedicine, antimicrobial resistance, Indian flora, precision nanomedicine, cancer nanomedicine, metal nanoparticles, biogenic nanoparticles

## Abstract

Biogenic metal nanoparticles are naturally covered with the phytochemical corona, which includes plant-derived metabolites. Emerging evidence suggests that the phytochemical corona, together with the intrinsic properties of the metallic core, contributes significantly to the biological identity, therapeutic behavior, and safety profile of biogenic nanoparticles. In this review, we go beyond the traditional view of plant extracts as reducing and capping agents to the phytochemical corona as a programmable nano–bio interface. Green synthesis from Indian flora has potential that can yield coronas rich in flavonoids, polyphenols, terpenoids, and alkaloids. Each corona composition contributes to different physicochemical properties, such as cellular interactions and downstream effects on reactive oxygen species, endocytic uptake and signaling pathways (p53, AKT, MAPK). When in contact with biological fluids, the corona adsorbs host proteins, giving rise to a hybrid interface that further influences the therapeutic outcome. The corona composition directly contributes to the biological activities of these nanoparticles: for example, anticancer, antimicrobial, antioxidant, and antiparasitic. The corona offers intrinsic targeting, stimuli-responsive release and improved stability for drug delivery. Toxicity and safety assessment shows dose-dependent effects, organ accumulation and long-term concerns for which standardized testing is needed. Translational challenges include: reproducibility, seasonal and geographic phytochemical variation, variability in extraction methods, scalability, shelf life and regulatory ambiguity. Future directions include Artificial intelligence (AI)-driven phytosynthesis, precision nanomedicine, nano–bio interface engineering, multi-omics integration, exploration of endangered Indian flora, and digital twin modeling. This review provides a roadmap for engineering phytochemical coronas as precision nanomedicine platforms by shifting the focus from core to corona and from empirical recipes to predictive design. It positions biogenic nanoparticles not only as eco-friendly alternatives, but as programmable, superior therapeutics for cancer and drug-resistant infections.

## 1. Introduction

Although some synthetic nanoparticle formulations have attained clinical success, the translational efficiency of many nanomedicine platforms is still hampered by issues related to protein corona formation, heterogeneous bio-distribution, immune recognition, and off-target accumulation [1,2,3]. Despite the regulatory approval of more than 80 nanomedicines, studies indicate that less than 1% of systemically administered nanoparticles accumulate at the intended disease site, highlighting persistent barriers to effective clinical translation [2]. Recent analyses further emphasize that the gap between preclinical success and clinical approval remains substantial, with most nanomedicine candidates failing during translational development due to insufficient efficacy, safety concerns, and manufacturing challenges [4,5].

Biogenic synthesis mediated by plants has been developed as a sustainable alternative. The field has remained within a reductionist vision. Plant extracts are believed to be only reducing and capping agents [4]. During synthesis, a phytochemical corona forms on the surface of the nanoparticle. This corona is a multilayer shell of complicated bioactive metabolites. It provides an inherent, programmable biological function. Functionality is lacking in chemically synthesized counterparts [5]. Synthetic nanoparticles need to be functionalized with targeting ligands and stealth polymers. Biogenic nanoparticles are produced with a functional biological interface already in place [6]. The phytochemical corona is a paradigm shift in nano–bio interfaces from passive to active [7].

In biological fluids, the protein corona is formed in an uncontrolled way. It often leads to immune clearance. The phytochemical corona is designed in the synthesis process. It enables deterministic control of the identity of the nanoparticles [8]. This changes how the nanoparticles interact with cells, how they evade the immune system, how they cross biological barriers and how they deliver therapeutic payload [9]. Phytochemical coronas reduce macrophage uptake by as much as 70% when compared to bare nanoparticles. The tumor uptake is increased threefold in murine xenograft models [10]. The basic principles of how specific phytochemical landscapes from *Azadirachta indica* versus *Ocimum tenuiflorum* versus *Withania somnifera* govern corona architecture, cellular recognition and downstream molecular logic are largely unknown [11]. This does not permit rational design of biogenic nanoparticles. This is the biggest roadblock for clinical translation [12]. The field is based on empirical trial and error. Every research group uses different extraction protocols, synthesis conditions and characterization methods. Cross-study comparison and meta-analysis is virtually impossible [13].

India is home to more than 45,000 documented plant species. Of these, more than 7000 are documented medicinal plants in the traditional Ayurvedic, Siddha and Unani systems. This offers an unparalleled natural laboratory to decode the nano–bio grammar [14]. The Western Ghats itself is a World Heritage Site of UNESCO. It has more than 5000 endemic plant species. Many of them have never been studied for the synthesis of nanoparticles [15]. Regional climatic variations across this biodiversity hotspot led to different phytochemical fingerprints. This allows for systematic structure activity relationship (SAR) analysis between corona composition and therapeutic function [16]. India’s nanotechnology infrastructure converges with its biodiversity. The Nano Mission was launched in 2007 with funding of more than Rs 1000 crore. More than 50 dedicated nanobiotechnology centers exist. The National Nanotechnology Mission has been launched for 2024–2030. This convergence allows for translational innovation [17]. High throughput screening facilities have been established in several Indian institutions. These facilities process hundreds of plant extracts per week. They estimate the efficiency of the synthesis of nanoparticles and biological activity [18]. The antimicrobial resistance (AMR) crisis is estimated to cause 1.27 million deaths a year directly and is expected to reach 10 million by 2050. That is more than cancer deaths. New therapeutic approaches are needed [19]. Biogenic nanoparticles have a multi-target mechanism of action. They provide a means of bypassing normal resistance pathways [20]. Traditional antibiotics usually have a single bacterial target. Phytochemical corona nanoparticles simultaneously disrupt membranes, generate oxidative stress, inhibit efflux pumps, degrade biofilms and interfere with quorum sensing. This makes the development of resistance statistically very unlikely [21]. Cancer cells are highly heterogeneous. This happens both across patients and within individual tumors. Many targeted therapies have failed [22]. Phytochemical corona nanoparticles demonstrate multi-target multi-pathway action. This activity is in line with the concept of polypharmacology. One agent targets multiple disease relevant targets. This lowers the likelihood of resistance development [23]. Recent single-cell sequencing studies show inhibition of proliferation of p53 mutant, KRAS mutant and HER2 amplified breast cancer cells by biogenic gold nanoparticles. Potency is similar among these subtypes (IC50 8–15 µg/mL). Targeted agents are effective only in selected molecular subtypes [24].

The concept of phytochemical corona is inspired by the protein corona paradigm. It has some unique benefits for therapeutic applications [25]. The protein corona is stochastic and patient-dependent. Often harmful, it masks target ligands, triggers hypersensitivity reactions, and accelerates clearance. The phytochemical corona is reproducible and deterministic. It is intrinsically healing [26]. The inherent bioactivity is attributed to the surface-bound plant secondary metabolites. Flavonoids inhibit efflux pumps nanomolar potency. Terpenoids break down microbial cell membranes. Polyphenols inhibit NFκB to modulate inflammatory signaling [27]. The phytochemical corona is not a passive coat. It is an active signaling entity that constantly interacts with the biological environment [28]. Surface-bound phytochemicals interact with cell surface receptors, activate specific endocytic uptake pathways, and trigger intracellular signaling cascades independent of the nanoparticle core [29]. This shifts the design paradigm from core-centric to corona-centric. It has important implications for the development of nanotherapeutics and regulatory assessment [30].

This article is a narrative review to summarize and critically discuss the emerging role of phytochemical coronas in biogenic metal nanoparticles and their applications in precision nanomedicine. This review will be of interest to researchers in the fields of nanomedicine, nanobiotechnology, pharmaceutical sciences, cancer biology, microbiology, and translational medicine who have an interest in the emerging concept of phytochemical corona engineering.

## 2. Green Synthesis as a Corona Engineering Platform

### 2.1. Phytosynthesis from a Rational Design Perspective

Green synthesis has traditionally been promoted as an environmentally sustainable alternative to conventional nanoparticle fabrication methods because it minimizes the use of hazardous chemicals and energy-intensive processes [31]. However, the significance of plant-mediated nanoparticle synthesis extends beyond its ecological advantages. Increasing evidence suggests that the diverse array of phytochemicals present in plant extracts can influence nanoparticle nucleation, growth, morphology, and surface characteristics, thus contributing to the formation of biologically relevant interfaces [32]. Before functioning solely as reducing and stabilizing agents, phytochemicals may play an important role in defining the physicochemical properties of biogenic nanoparticles.

Several reaction parameters, including pH, temperature, precursor concentration, and light exposure, have been reported to impact nanoparticle synthesis and the resulting surface chemistry [33]. Even though complete control over corona composition remains challenging because of the intrinsic variability of plant extracts, understanding the relationship between synthesis conditions and nanoparticle characteristics may enable more reproducible and rational approaches to phytochemical corona engineering.

### 2.2. Contributions of Phytochemicals to Nanoparticle Formation

The formation of plant-mediated nanoparticles is a complex process involving the coordinated action of multiple phytochemical classes [34]. Polyphenols and flavonoids are recognized for their electron-donating capabilities, enabling reduction in metal ions while also contributing to nanoparticle stabilization [35]. Terpenoids, alkaloids, proteins, and polysaccharides may further influence crystal growth, surface charge characteristics, and colloidal stability [36]. Because the phytochemical composition varies substantially among plant species, nanoparticles synthesized from different botanical sources often exhibit distinct physicochemical and biological properties. Still, the precise contribution of individual phytochemicals to these outcomes remains incompletely understood and warrants further investigation [37].

### 2.3. Mechanisms of Phytochemical Corona Formation

Phytochemical corona formation occurs simultaneously with nanoparticle synthesis and represents a dynamic process rather than a discrete event [38]. Initially, metal ions such as Ag^+^ and Au^3+^ interact with functional groups present in phytochemicals, including hydroxyl, carbonyl, and amino moieties [39]. These interactions facilitate the reduction in metal ions to their zero-valent state through electron transfer mechanisms involving compounds such as polyphenols, quinones, and enzymatic reductases [40].

Later, reduced metal atoms aggregate to form nanoscale nuclei that serve as templates for further growth [41]. During this stage, phytochemicals begin to adsorb onto the nanoparticle surface through both covalent and non-covalent interactions, contributing to the development of a relatively stable surface-associated layer [42]. Following exposure to biological environments, additional interactions with proteins and biomolecules may further modify this interface, resulting in a dynamic nano–bio boundary that influences biological behavior [43]. Understanding these mechanistic processes is essential for improving reproducibility and predicting therapeutic performance.

### 2.4. Factors Influencing Phytochemical Corona Composition

Variability in nanoparticle synthesis remains one of the major challenges in the field of biogenic nanomedicine [44]. Several reaction parameters have been shown to influence both nanoparticle properties and phytochemical corona composition. For example, pH affects the ionization state of phenolic compounds, thereby influencing surface charge and colloidal stability [45]. Temperature alters reduction kinetics and crystallization processes, potentially affecting nanoparticle morphology and surface defect formation [46]. Light-assisted synthesis has also been reported to modulate nanoparticle size distribution through photocatalytic reduction pathways involving photoexcited electron transfer [47].

Systematic investigation of these variables may contribute to the development of standardized synthesis protocols. Moving beyond empirical optimization toward evidence-based design strategies will be important for improving reproducibility and facilitating clinical translation [48].

### 2.5. Plant-Dependent Morphological Diversity

Plant species differ considerably in their phytochemical profiles, which may contribute to variations in nanoparticle morphology and biological properties [49]. For instance, extracts from *Murraya koenigii* have been reported to predominantly generate quasi-spherical silver nanoparticles [50], whereas *Carica papaya* extracts have been associated with the formation of anisotropic nanostructures, such as triangular nanoprisms [51]. These differences may arise from selective interactions between phytochemicals and specific crystallographic facets during nanoparticle growth [52].

Although emerging evidence suggests that phytochemical composition influences nanoparticle morphology, predictive relationships between plant-derived metabolites and structural outcomes remain insufficiently categorized. Integrating phytochemical profiling with advanced nanoparticle characterization techniques may enable a more comprehensive understanding of these associations [53]. Because nanoparticle size and shape can affect cellular uptake pathways and intracellular trafficking, further studies are required to determine how morphological diversity influences therapeutic responses [54].

### 2.6. The Phytochemical Corona as a Functional Nano–Bio Interface

The phytochemical corona should not be considered merely a passive capping layer generated during nanoparticle synthesis [55]. Instead, it represents an important component of the nano–bio interface that may influence nanoparticle stability, interactions with biological fluids, cellular recognition, and therapeutic activity [56]. Surface-associated phytochemicals can contribute to resistance against aggregation and may modulate protein adsorption following exposure to physiological environments [57].

Thus, characterization of corona composition, thickness, and dynamic behavior is essential for understanding the biological identity of biogenic nanoparticles. Recognizing the phytochemical corona as a functional interface rather than a synthesis byproduct may support the development of more rational approaches to nanoparticle design [1].

### 2.7. Potential Advantages and Current Limitations of Biogenic Nanoparticles

Biogenic nanoparticles offer several potential advantages compared with conventionally synthesized systems, including environmentally sustainable production processes and the presence of naturally derived surface functional groups [58]. Some studies have reported enhanced antimicrobial activity, altered cellular uptake profiles, and modified immune interactions associated with phytochemical-coated nanoparticles [59]. Yet, these observations are often context-dependent and influenced by factors such as nanoparticle composition, synthesis methodology, and experimental conditions.

Therefore, it is premature to conclude that biogenic nanoparticles are universally superior to their chemically synthesized counterparts. Both approaches possess distinct advantages and limitations, and direct comparative studies using standardized experimental frameworks are needed to determine their relative therapeutic potential.

### 2.8. Existing Bottlenecks and Future Directions

Despite significant progress, several challenges continue to limit the advancement of phytochemical corona-mediated nanomedicine. These include the lack of high-throughput analytical platforms for corona characterization, the absence of standardized phytochemical reference libraries, and limited quantitative models linking synthesis parameters to biological outcomes [60]. Furthermore, seasonal and geographical variations in plant metabolite composition can contribute to batch-to-batch variability, thereby affecting reproducibility and scalability.

Future research should focus on establishing standardized reporting guidelines, integrating advanced analytical techniques for corona characterization, and developing predictive computational models to support rational nanoparticle design. Controlled cultivation systems, including hydroponic approaches, may also help reduce variability in source materials and improve manufacturing consistency.

### 2.9. Opportunities Arising from India’s Biodiversity

India possesses exceptional botanical diversity, mainly within biodiversity hotspots such as the Western Ghats and Eastern Himalayas, which contain numerous medicinal plant species with largely unexplored nanobiotechnological potential [61]. Plants including *Tinospora cordifolia*, *Emblica officinalis*, and *Withania somnifera* have demonstrated promising biological activities when employed in nanoparticle synthesis [62,63].

In combination with national initiatives supporting nanotechnology and biotechnology research, this biodiversity offers valuable opportunities for developing comprehensive phytochemical nanoparticle libraries and investigating relationships between phytochemical composition and biological function [64]. Continued interdisciplinary collaboration among botanists, chemists, material scientists, and biomedical researchers will be essential for translating these discoveries into clinically relevant applications.

## 3. Nano–Bio Interface and Phytochemical Corona

### 3.1. Illusion of Naked Nanoparticles

There is a fundamental misconception in the literature of nanomedicine. The metallic core [57] is believed to dictate the biological identity of NPs. In reality, nanoparticles are coated with a layer of biomolecules within milliseconds of their exposure to any biological milieu. This modifies their interfacial properties [65]. For biogenic nanoparticles, the realization is deeper. They are never empty. They have a preformed phytochemical corona from the time of synthesis [66].

This preformed corona serves as a biological passport. It governs the protein adsorption profiles, cell recognition, immune escape and therapeutic destiny [67]. The mass is provided by the core and the specificity is provided by the corona [25].

Quantitative proteomics studies reveal that the corona composition on biogenic nanoparticles is dramatically different from that on chemically synthesized controls [68]. Interestingly, this unique corona is rich in apolipoproteins, especially apolipoprotein A I, which promotes LDL receptor-mediated targeting, while the abundance of opsonins (fibrinogen and immunoglobulins) is dramatically reduced, thus conferring stealth properties and longer circulation time [69,70].

### 3.2. Phytochemical Corona as a Preexisting Biological Identity

The classical protein corona paradigm describes the interaction of nanoparticles with biological fluids upon administration [5]. Plant-mediated nanoparticles already have a phytochemical corona. This changes the interaction in a fundamental way [71]. The protein corona is passive and detrimental, leading to immune clearance and masking of targeting ligands. The phytochemical corona is a dynamic programmable interface [1].

Biogenic nanoparticles must be reinterpreted as the first nano constructs of corona. The core is primarily a scaffold for the display of phytochemicals [71]. The corona is responsible for the therapeutic and targeting functionalities and the core is responsible for structural integrity, imaging and as a reservoir for metal ions [72,73].

### 3.3. Hard and Soft Corona

The phytochemical corona is hierarchical and consists of a stable inner corona (hard corona) and an exchangeable outer corona (soft corona). The hard corona is responsible for long-term colloidal behavior and shows higher-affinity interactions, whereas the soft corona is characterized by a more dynamic exchange with the surrounding environment [74].

The hard and soft coronas can dynamically exchange, allowing the nanoparticle to adapt and sense the environment, something that is not possible with chemically synthesized nanoparticles [75]. In the acidic pH of endosomes, soft corona components may desorb, leading to drug release or exposing membrane-active hard corona components that enhance endosomal escape [76].

### 3.4. Physicochemical Modulation

The phytochemical corona is important in defining the basic physicochemical behavior of biogenic nanoparticles [71]. This dynamic coating layer can increase the effective hydrodynamic diameter of the nanoparticle, which influences its biological fate [77,78]. Additionally, the corona makes the nanoparticle surface zeta potential become more negative, which enhances the colloidal stability due to the increased electrostatic repulsion [71,79]. The ultra-thick surface coating offers good steric stabilization, effectively preventing particle aggregation and providing long-term colloidal stability even in challenging conditions [71,79]. Such physicochemical changes then increase the shelf stability and serum compatibility of the nanoparticle, which leads to an increase in its circulation half-life in the body [77,78].

### 3.5. Uptake of Cells

The corona composition regulates the mechanism and efficiency of nanoparticle internalization [80]. Phytochemical-rich coronas promote clathrin-mediated endocytosis through interactions with cell surface glycoproteins and lectins [81]. High-flavonoid coronas activate caveolae-mediated pathways that lead to transcytosis and inhibit lysosomal degradation [82]. The corona can also engage receptor-mediated endocytosis: some phytochemicals mimic natural ligands. Quercetin mimics epidermal growth factor, binds to EGFR and triggers uptake [83].

### 3.6. Immunomodulation

The phytochemical corona has two immunomodulatory functions [84]. Firstly, it functions as a stealth coating, reducing opsonin adsorption and macrophage recognition through the CD47–SIRPα axis, thus prolonging circulation time [85]. Second, some phytochemicals actively modulate cytokine profiles: they inhibit pro-inflammatory TNF α and IL 6 and promote anti-inflammatory IL 10 [86].

### 3.7. Corona Hybrid

When entering the blood, the phytochemical corona adsorbs host proteins and thus a hybrid corona is formed [66]. Polyphenol-rich coronas preferentially bind albumin and apolipoproteins, excluding fibrinogen, allowing for LDL receptor-mediated targeted delivery [87]. The hybrid corona is the true biological identity of biogenic nanoparticles in vivo [5].

### 3.8. Intracellular Signaling

The corona induces reactive oxygen species (ROS) generation, activates the p53 pathway, inhibits PI3K/AKT, and modulates the MAPK cascade [88]. The p53 activation induces cell cycle arrest by p21 and therefore affords a therapeutic window for deoxy ribose nucleic acid (DNA)-damaging agents while protecting normal cells [89].

### 3.9. Apoptosis, Autophagy

The corona can induce both autophagy and apoptosis and the net cell death is determined by the balance of the two [90]. Autophagy is an adaptive response to mild oxidative stress. In this case, the balance is shifted towards apoptosis when ROS production exceeds autophagic capacity [91].

### 3.10. Reducing Toxicity

The phytochemical corona reduces toxicity via three mechanisms [92]. First, it keeps the reactive metal core from direct contact with cellular membranes [93]. Second, antioxidant phytochemicals scavenge the excess ROS and thus maintain the redox homeostasis [94]. Third, the corona modulates metal ion leaching: chelation of surface metal atoms by catechol groups slows down dissolution and prevents toxic ionic bursts [95].

### 3.11. Plant-Specific Corona Diversity

Each plant species makes its own corona [96]. *Azadirachta indica* produces limonoids and its crown inhibits NF κB [97]. *Ocimum sanctum* produces eugenol and its corona mediates caveolae-dependent uptake [98]. *Withania somnifera* produces withanolides, and its corona activates p53 [99]. *Emblica officinalis* gives tannins. Its corona has ultrahigh antioxidant activity [100]. *Tinospora cordifolia* produces cordifolioside, and its corona shows broad spectrum antimicrobial activity [101].

### 3.12. The Interface of Drug Delivery

The protein corona is an active interface for drug delivery [102]. Its various functional groups allow the loading of therapeutic agents by covalent or non-covalent interactions [1]. Moreover, the corona provides stimuli-responsive drug release; for example, the pH-sensitive linkages in its structure enable drug release in the acidic endosomes, and the redox sensitive disulfide bonds exploit the intracellular glutathione gradients to trigger payload delivery [103,104].

### 3.13. Characterization of Phytochemical Corona Composition

Various technologies have been developed for characterization of phytochemicals derived from variety of plants (Table 1), however, identification of bioactive metabolites in plant extracts alone is insufficient; direct evidence demonstrating their incorporation into the nanoparticle corona is essential.

## 4. Biological Activities of Biogenic Nanoparticles

The schematic shows the multistep molecular cascade by which biogenic nanoparticles functionalized with phytochemical coronas induce cancer cell death. The process involves the formation of phytochemical corona on the metal/metal oxide nanoparticle cores (Ag, Au, ZnO, CuO, Se) using bioactive metabolites (flavonoids, polyphenols, terpenoids, alkaloids, proteins, sugars) of Indian medicinal plants. Cellular uptake triggers two interrelated death pathways: (i) ROS-mediated mitochondrial apoptosis that includes generation of oxidative stress, loss of mitochondrial membrane potential, increased permeability transition pore opening, cytochrome c release and caspase dependent apoptosis and (ii) DNA damage-mediated cell cycle arrest that includes DNA strand breaks, base modifications, oxidative lesions, and subsequent upregulation of p53, p21 and p27, leading to G1/S or G2/M checkpoint activation. The junction of these pathways guarantees synchronized and non-cross resistant elimination of cancer cells, establishing phytochemical corona nanoparticles as the next generation of superior anticancer therapeutics.

**Figure 1:**
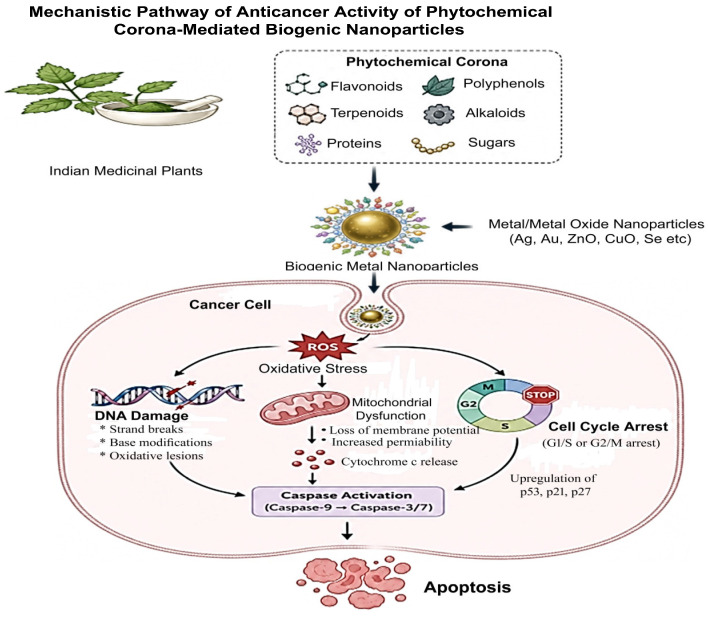
Mechanistic pathway of anticancer activity of phytochemical corona-mediated biogenic nanoparticles. (Source: BioRender.com).

As shown in Figure 1, biogenic nanoparticles target cancer cells by dual activation of the two core death pathways, mediated by their phytochemical corona: ROS-driven mitochondrial apoptosis and DNA damage-induced cell cycle arrest. This coordinated multi-pathway disruption makes the emergence of resistance much less likely and positions phytochemical corona nanoparticles as a strategic next-generation platform for precision oncology [105].

### 4.1. Anticancer Mechanisms

Biogenic nanoparticles force cancer cells to undergo an irreversible death program [106]. The natural coating on these nanoparticles, often called the phytochemical corona, makes it possible for them to attack cancer cells in several different ways at once, unlike conventional chemotherapy drugs that usually only target one thing [107]. None of these pathways cross-talk, which is very important in heterogeneous tumors, where different sub-clones often resist any single drug [107].

The key mechanism is oxidative stress. Biogenic nanoparticles exceed the cell’s capacity to mop up ROS, forcing the cell beyond its tolerance [108]. This is supplemented by the phytochemical corona, which enhances redox cycles through polyphenols and flavonoids attached to the nanoparticle surface [109]. These compounds donate one electron to generate semiquinone radicals, which further reduce oxygen to superoxide, leading to even more ROS [110].

All that oxidative damage affects the mitochondria through depolarization of the inner membrane, opening of the permeability transition pore, cessation of ATP prodution, and leakage of proapoptotic factors into the cytoplasm [110]. Release of cytochrome c leads to formation of the apoptosome and activation of the intrinsic apoptosis pathway. The initiator caspase 9 lights up, and then the executioner’s caspase 3 and caspase 7 start cutting up PARP, lamins, and cytoskeletal proteins [111]. The cell is basically taking itself apart.

Biogenic nanoparticles can also cause direct DNA damage, leading to single-strand breaks, double-strand breaks, and oxidative lesions in DNA bases [112]. The cell’s DNA damage response is activated and halts the cell cycle at either the G0/G1 or G2/M checkpoint, preventing any cell with damaged DNA from replicating and spreading the damage [113].

Nanoparticles activate a few important pathways at the molecular level that lead to apoptosis [107]. One protein, PUMA, acts as a central hub [114]. It binds and inactivates the anti-apoptotic members of the BCL 2 family, and that tips the balance towards mitochondrial outer membrane permeabilization—the point of no return for the cell [114]. At the same time, nanoparticles have been shown to activate p53 pathway [115]. Once active p53 drives transcription of several proapoptotic genes including PUMA itself, BAX, NOXA and the cell cycle inhibitor p21 [115]. Nanoparticles can also blunt the PI3K/AKT survival pathway [116]. When PI3K/AKT is down, FOXO transcription factors are released and are free to drive the cell toward apoptosis [116]. Furthermore, nanoparticles modulate the MAPK network, especially JNK and p38, and these kinases induce cell death through the phosphorylation of proteins such as c Jun and ATF2 [117]. Since all these pathways lead to the same goal, nanoparticles attack cancer cells from different angles at the same time [105]. None of those routes cross each other [118]. That is why acquired resistance is very unlikely. In long-term selection experiments, cancer cells never developed stable resistance to biogenic gold nanoparticles, while resistance to doxorubicin appeared within eight weeks [118].

### 4.2. Mechanism of Antimicrobial Action

Biogenic nanoparticles are efficacious against a broad spectrum of microbes including Gram-positive bacteria, Gram-negative bacteria, fungi and even viruses [119]. Their principal weapon is membrane disruption [120]. Nanoparticles bind to negatively charged lipopolysaccharides (Gram-negative bacteria) or lipoteichoic acids (Gram-positive bacteria) [120]. This binding thins the membrane, punches holes in it and makes the cell ooze its contents [120]. Nanoparticles also produce ROS, which also contribute to the killing effect through damage to proteins, lipids and DNA [121].

Superoxide inhibits iron–sulfur cluster enzymes, thus blocking energy metabolism [122]. Hydroxyl radicals are even less selective, oxidizing almost any biomolecule they encounter [123]. Nanoparticles [122] also cause protein unfolding. They bind to thiol groups of important enzymes, altering the shape of the enzyme and preventing it from working [122]. Nanoparticles also cause direct damage to DNA, preventing replication and transcription, leading eventually to cell death [124]. Biogenic nanoparticles can also overcome drug resistance by blocking efflux pumps [125]. They inhibit antibiotic efflux by targeting the substrate-binding sites of RND and ABC transporters. This restores the internal antibiotic concentration to effective levels [125]. Experiments with ethidium brmide showed that pump overexpressing strains held the dye three to ten times longer after exposure to nanoparticles, a clear indication that efflux is blocked [126]. Furthermore, nanoparticles induce oxidative stress, which down-regulates the genetic expression of eflux pump genes [127]. This results in a long term reversal of resistance, rather than a temporary block [127]. Biofilms are not spared either [128]. Biogenic nanoparticles peetrate the biofilm matrix, degrade the extracellular polymeric substances and disrupt quorum sensing [128]. Finally, the combination of nanoparticles with conventional antibotics shows a synergistic effect [129]. Minimunm inhibitory concentrations (MICs) can be reduced up to 90% and the combination regains activity versus multidrug resistant clinical isolates [130].

### 4.3. Antioxidant and Redox Modulation

Biogenic nanoparticles have inherent antioxidant ability because of the plant cheicals packed onto their surface. They scavenge toxic molecules: superoxide, hydrogen peroxide, hydroxyl radicals and peroxynitrite.

The mild stress caused by the nanoparticle surface activates the Nrf2 pathway, which causes an increase in protective enzymes such as HO 1, NQO1, GST, catalase and SOD [130]. Depending on the cell type, these nanoparticles behave in two opposite ways.

In normal cells, they are anti-oxidants, but they work as pro-oxidants in cancer cells [130]. This selectivity is a consequence of the two cell types having different redox statuses. Cancer cells inherently work with higher oxidative stress levels. So, they are more vulnerable to an extra oxidative hit. Normal cells can take that same hit and not be hurt. This offers a therapeutic window as a distinct advantage over conventional cheotherapy [131].

### 4.4. Antiparasitic Activity

Biogenic nanoparticles are effective against several protozoan parasites such as *Plasmodium*, *Leishmania* and *Trypanosoma* [132]. Their main target is an enzyme known as trypanothione reductase. This enzyme is specific to these parasites and is essential for their redox balance [133]. Flavonoids and terpenoids attached to the nanoparticles inhibit the enzyme. This results in depletion of the reduced trypanothione, leading to lethal oxidative stress within the parasite [134]. Biogenic nanoparticles simultaneously inhibit the topoisomerase enzymes. This stops the parasite from making new DNA and copying its genes [135]. These nanoparticles hit a number of key processes simultaneously, making it very difficult for the parasite to become resistant [136].

## 5. Nanoparticles and Antimicrobial Resistance (AMR)

### 5.1. Modulation of Transporters by Drugs

Biogenic nanoparticles inhibit drug efflux pumps such as P-glycoprotein (P-gp) in three ways, i.e., by binding to the drug pocket, changing membrane fluidity, and shutting off the gene that encodes the pump [137]. This triple action brings antibiotic levels back inside bacteria and reverses resistance to macrolides, fluoroquinolones and tetracyclines. Polyphenols like curcumin, quercetin and EGCG on the surface of the nanoparticles also inhibit MRP1, a pump that exports glutathione-conjugated drugs. These phytochemicals also inhibit another efflux pump called BCRP which works on methotrexate and fluoroquinolones. The reported synergistic effects of blocking P-gp, MRP1 and BCRP together are said to reduce the amount of antibiotic needed by 50–70% [137]. Biogenic nanoparticles also stimulate uptake pumps known as SLC transporters (e.g., OCT1 and MATE1) that shuttle cationic drugs into the cell [138]. These nanoparticles are more efficient than conventional inhibitors for the reversal of multidrug resistance through blocking efflux and enhancing uptake [138].

### 5.2. Efflux Pump Inhibitors

Bacteria often develop resistance to antibiotics by using protein pumps in their cell walls to expel the drugs [139]. Biogenic nanoparticles are effective against many types of these drug pump systems and act in very different ways to older, synthetic inhibitors. One prominent way is that the natural polyphenols coating the nanoparticles can directly interfere with the activity of the pumps [139]. These pumps can also be permanently turned off by a type of oxidative stress induced by green-synthesized nanoparticles. The big advantage is that these nanoparticles attack pumps on multiple pathways simultaneously, and bacteria are much less likely to develop resistance towards them [140].

### 5.3. Disruption of Biofilms

A biofilm is a community of bacteria that stick to a surface and form a slimy matrix that provides them with a high degree of antibiotic resistance [141]. Biogenic nanoparticles can penetrate biofilm matrices, owing to their nanoscale dimensions and surface properties. However, plant mediated nanoparticles frequently exhibit negative zeta potential values due to surface-associated phytochemicals, although surface charge characteristics may vary depending on synthesis conditions and corona composition. Once inside, they block the bacteria’s communication system called quorum sensing, which normally sends the command to start building the biofilm [142]. The nanoparticles attack the biofilm by destroying the vital components of the matrix, such as DNA and proteins. This breakdown also exposes previously hidden bacteria to the immune system, making them easier for our own cells to clear out [143]. Even better, by destroying the biofilm, regular antibiotics can now reach and kill the bacteria with doses up to 100 times lower. These features make biogenic nanoparticles very promising in the treatment of persistent chronic infections like “*Pseudomonas aeruginosa*” in cystic fibrosis or bacteria on catheters and joint implants [142].

### 5.4. Interference with the Resistance Pathway

The interference of biogenic nanoparticles with several bacterial defense pathways at the same time makes it very difficult for resistance to evolve [144]. Reactive oxygen species are generated by nanoparticles that damage beta lactamase enzymes (both the serine and metallo types) so that they cannot break down penicillin-like antibiotics. These nanoparticles also inhibit aminoglycoside modifying enzymes and chloramphenicol acetyltransferases, restoring activity against gentamicin and chloramphenicol [144]. Nanoparticles intercalate into DNA or induce oxidative lesions, preventing the transfer of resistance plasmids between bacteria and reducing the spread of genes like _bla_NDM and mcr 1 [144]. Experiments over long periods of time show that bacteria do not develop stable resistance to biogenic nanoparticles, while resistance to conventional antibiotics such as ciprofloxacin increases hundreds of times [137]. Lastly, nanoparticles decrease the virulence factors and capsule production of bacteria, making them easier to kill by the immune system [138].

## 6. Drug Delivery Applications

Nanoparticles would deliver drugs to diseased tissues, increasing efficacy and reducing side effects. One way they do this is through the “enhanced permeability and retention” (EPR) effect: tumors have leaky blood vessels and poor drainage and so nanoparticles tend to accumulate there [145]. Alternatively, active targeting can be used, in which ligands such as folic acid or antibodies are attached to the surface of the nanoparticle, which can then only bind to receptors that are over-expressed on cancer cells [131]. Biogenic nanoparticles have an inherent advantage their natural phytochemical corona already provides targeting groups without the need for synthetic chemical steps. Scientists often try to coat nanoparticles with polyethylene glycol (PEG) in order to have them circulate longer in the blood, a process called PEGylation [131]. The PEG coating acts as a “stealth” shield to prevent the immune system from quickly clearing the particles away [146]. A limitation with PEGylation is that too much PEG can actually block the nanoparticles from getting into cells, so the density must be properly adjusted [146]. Antibodies can be chemically conjugated to nanoparticles in order to achieve active targeting, and “immunonanoparticles” are made that look for specific antigens on cancer cells [131]. The natural carboxyl, amine and hydroxyl groups on the surface of biogenic nanoparticles allow the efficient conjugation of antibodies and other ligands without the need of harsh chemistry [147]. Biogenic nanoparticles have a powerful feature of “stimuli-responsive” drug release, which allows them to release their cargo only in a particular condition [147]. For example, pH-sensitive chemical bonds are broken in the acidic environment of tumors or endosomes (pH 5.0–6.5) but stable in a normal body pH of 7.4, or the high concentration of glutathione inside cells (100–1000× higher than outside) can be used in redox-sensitive disulfide bonds to trigger release only after the nanoparticle has entered the target cell [147]. Enzyme cleavable peptide linkers may also be used, which are cleaved by matrix metalloproteinases that are over-active in tumors and inflamed tissues. The phytochemical corona can also vary with pH, which is related to the rate of drug release [148], and this is another level of control. Perhaps the most elegant strategy is “corona driven targeting”: the corona’s complex of plant polyphenols and adsorbed serum proteins directs the nanoparticle to specific cells (e.g., cancer cells that over-express LDL receptors) without any synthetic ligand added [148].

## 7. Safety and Toxicity Evaluation

### 7.1. Dose-Dependent Toxicity

Biogenic nanoparticles have a steep dose response relationship for toxicity. A complete characterization of this relationship is required for safe clinical use [149]. At low doses (1–10 μg/mL for silver nanoparticles, 10–100 μg/mL for gold nanoparticles), the cells function normally. This is done through Nrf2-mediated antioxidant responses with upregulation of protective enzymes like heme oxygenase-1, catalase and superoxide dismutase. Cells also depend on autophagic clearance through the LC3-II/beclin-1 pathway [150].

At intermediate doses (10–50 μg/mL for silver, 50–200 μg/mL for gold), ROS are transiently generated, which induce adaptive stress responses. These include upregulation of heat shock proteins and activation of DNA repair pathways. Importantly, they occur without committing the cell to death [151]. At high doses (>50 μg/mL for silver, >200 μg/mL for gold), ROS production exceeds the cell’s antioxidant capacity. Glutathione depletion exceeds 80%. Decreased mitochondrial membrane potential is >50%. A 3–20-fold increase in caspase activity leads to irreversible cellular damage [152]. Identifying the therapeutic window, the dose range where the efficacy is greater than toxicity, is a critical step for clinical translation [149].

### 7.2. Accumulation in Organ

The distribution of the nanoparticles in the body after injection depends on factors such as size, charge and corona composition. The mononuclear phagocyte system (MPS) consists of the Kupffer cells of the liver and the macrophages of the spleen, and it captures most of the nanomedicines [153]. This MPS at high doses can cause liver damage, as evidenced by increases in ALT/AST enzymes and inflammation in the tissue [154]. Changes in tissue organization and cell death can also occur in the spleen [154]. Very small nanoparticles (<8 nm) can pass through kidney filters and be present in the urine within hours. Most nanoparticles are unlikely to reach the brain but a protein coat rich in ApoE can help them cross the blood–brain barrier [155]. The uptake of nanoparticles by the liver and spleen can be reduced by up to 80% when coated with PEG [156]. The “stealth” layer of PEG remains on the surface of the particles, allowing for longer circulation times and less off-target accumulation.

### 7.3. Blood–Brain Barrier Crossing

The blood–brain barrier is a dense cellular layer that shields the brain and prevents most drugs from crossing it [157]. It is a major challenge for therapies to cross the blood–brain barrier by squeezing between cells or being ferried through cells, because of the tight seals and pumps that spit out foreign substances [157]. One of the major ways to bypass this barrier is through a process called caveolae-mediated transcytosis where the nanoparticles are actively transported across the cells [158]. Nanoparticles with specific properties, such as small size, positive surface charge or surface modification with specific molecules, such as transferrin, apolipoprotein-E or some flavonoids, can take advantage of this transcytosis pathway to enter into the brain [158]. The ability to cross the blood brain barrier is promising for the treatment of brain cancers such as glioblastoma and central nervous system infections, but it also raises questions about the long term safety in delicate tissues of the nervous system [159].

### 7.4. Safety Issues (Long Term)

The safety issues related to the chronic exposure and long-term retention of nanoparticles remain unresolved and need to be addressed before clinical translation [160]. Bioaccumulation can take place in the liver, spleen, bone marrow and lymph nodes, and for non-biodegradable nanoparticles (e.g., gold, silver, ceria and iron oxide), the retention half-lives are in the range of weeks to months [160]. Chronic toxicity in animal models after repeated or high dose administration is characterized by chronic inflammation (increased IL-6 and TNF-α), granuloma formation (aggregation of macrophages) and fibrosis [161]. Another concern is immunotoxicity caused by hypersensitivity reactions to phytochemical components or protein corona antigens and immunosuppression characterized by impaired dendritic cell maturation and reduced T-cell proliferation [162]. Some nanoparticle compositions have been shown to be genotoxic including DNA damage, chromosomal aberrations and micronuclei formation, raising concerns about carcinogenic potential and the need for long-term rodent bioassays [161]. Nanoparticle release during manufacturing, clinical use or disposal may result in environmental toxicity in aquatic organisms (zebrafish embryo toxicity, daphnia mortality) and soil microbiota, highlighting the urgent need for comprehensive long-term studies, including carcinogenicity bioassays, reproductive and developmental toxicity studies and life-cycle assessments, for regulatory approval [163].

## 8. Translation Challenges

### 8.1. Reproduction Issues

Indeed, the most serious obstacle to getting biogenic nanoparticles into the clinic is that different studies cannot reproduce each other’s results [164]. Even when scientists use the same plant species, the nanoparticles they get can be wildly different in size, shape, and surface charge. This variability is due to uncontrolled biological factors like the age of the plant, the tissue used, the local weather and even the soil composition. This idea is well summarized by the saying “same plant ≠ same nanoparticle” [164]. To make matters worse, different extraction methods, reaction conditions and ways of measuring the final product only add to the inconsistencies [165].

### 8.2. Seasonal and Territorial Variation

The chemistry of the plant extract is highly dependent on the season and the place of cultivation [166]. For example, the total phenolic content of *Ocimum sanctum* may range threefold, from 15 to 45 mg GAE/g depending upon the time of harvesting. The secondary metabolism of the plant is affected by the local temperature, the minerals in the soil, and the amount of rain that falls and the hours of sunlight. These environmental variables result in uncontrolled batch-to-batch variation, making it difficult to produce the same nanoparticles year-round or at different sites. One promising fix is controlled environment agriculture, in which plants are grown indoors under strictly managed conditions [166].

### 8.3. Variability of Extraction Method

Not only the chemical used but also the extraction method used dramatically changes the final nanoparticle [165]. Water extraction pulls out polar molecules, but leave lot of terpenoids behind. Ethanol extraction pulls out mid-polar molecules, but breaks down proteins. Other solvents concentrate different subsets of phytochemicals but they also have their own toxicity problems. Extraction temperature, time and solvent to plant ratio control what is extracted and how much. Currently, there is no standard extraction protocol, and it is therefore nearly impossible to compare studies [165].

### 8.4. Scalability Problems

The up-scaling from laboratory flask to industrial bioreactor for green nanoparticle synthesis is extremely difficult [167]. As the volume increases, problems such as non-uniform mixing, temperature gradients and poor mass transfer reduce the uniformity of the product. But at the scale of many tons, it becomes a logistical nightmare to get a consistent, standardized supply of high-quality plant biomass. The shape of the reactor, the stirring, and heat management all determine if the nanoparticles come out the same each time [167].

### 8.5. Shelf Life and Stability

Long term physicochemical stability is a critical prerequisite for the successful development and commercialization of biogenic nanoparticles. Stability influences not only product shelf life but also therapeutic efficacy, safety, and reproducibility during storage and administration. However, maintaining the stability of phytochemical corona mediated nanoparticles remains a significant challenge.

Over time, biogenic nanoparticles may undergo aggregation, oxidation, or alterations in their surface associated phytochemical corona, potentially affecting their biological activity and colloidal behavior [168]. Several environmental factors, including temperature, light exposure, pH, and ionic strength, have been shown to influence nanoparticle stability by modulating both the metallic core and the integrity of the phytochemical corona.

Various preservation strategies, such as freeze drying and spray drying in the presence of cryoprotectants or lyoprotectants (e.g., sugars and polyols), have been explored to improve storage stability. Although these approaches may prolong shelf life, they can also alter nanoparticle size distribution, surface properties, and redispersion characteristics upon reconstitution. Thus, the optimization of storage conditions and preservation methods is essential to maintain the desired physicochemical and biological properties of biogenic nanoparticles.

Current studies suggest that the shelf life of many biogenic nanoparticle formulations ranges from several weeks to a few months, which remains substantially shorter than the stability requirements expected for pharmaceutical products [168]. Therefore, systematic investigations aimed at improving long-term stability and establishing standardized storage protocols are necessary to support clinical translation.

### 8.6. Uncertainty in Toxicological Assessment

Although biogenic nanoparticles are frequently regarded as safer alternatives to conventionally synthesized nanomaterials because of their environmentally friendly production methods, important questions regarding their safety profile remain unresolved [169]. The term “green” synthesis should not be interpreted as an assurance of biological safety, and comprehensive toxicological evaluation remains essential.

Several studies have demonstrated that nanoparticles exhibiting minimal toxicity in *in vitro* cellular models may still induce adverse effects in vivo, particularly in organs involved in nanoparticle clearance, such as the liver, kidneys, and spleen. Furthermore, many currently available studies focus predominantly on short-term exposure scenarios and relatively high concentrations, whereas information regarding chronic exposure, bioaccumulation, immunotoxicity, reproductive toxicity, developmental toxicity, and carcinogenic potential remains limited [169].

Another major challenge is the lack of standardized testing protocols for evaluating the safety of biogenic nanoparticles. Differences in nanoparticle composition, phytochemical corona characteristics, experimental models, and exposure conditions hamper direct comparisons between studies. The development of harmonized toxicological assessment frameworks incorporating both short-term and long-term evaluations will therefore be essential to facilitate regulatory approval and ensure safe clinical implementation.

### 8.7. Regulatory and Standardization Challenges

Regulatory approval of nanomedicines requires robust evidence demonstrating manufacturing reproducibility, consistent physicochemical characteristics, and comprehensive safety and efficacy profiles. However, achieving these requirements remains particularly challenging for biogenic nanoparticles because of the inherent variability associated with plant-derived materials [164]. Variations in plant species, geographical origin, seasonal conditions, extraction procedures, and synthesis parameters can significantly influence nanoparticle characteristics and phytochemical corona composition. This variability complicates the establishment of standardized manufacturing protocols and quality control procedures. Moreover, unlike conventional pharmaceuticals, there are currently no internationally accepted pharmacopeial monographs or regulatory guidelines specifically addressing the production and characterization of plant-mediated nanoparticles [164].

An additional challenge relates to the regulatory classification of these materials. Depending on their intended application and composition, biogenic nanoparticles may be evaluated as nanomedicines, biologics, medical devices, or botanical products, each of which is governed by different regulatory frameworks. Furthermore, inconsistencies in characterization methodologies among laboratories limit reproducibility and complicate regulatory evaluation. Therefore, the development of standardized analytical methods and regulatory guidelines specifically tailored to biogenic nanomaterials represents a critical step toward successful clinical translation.

### 8.8. Commercialization Challenges

Despite the promising therapeutic potential of biogenic nanoparticles, several economic and technological barriers continue to impede their commercialization [170]. One of the major obstacles is the substantial variability associated with plant-derived starting materials, which necessitates extensive quality control measures and may reduce manufacturing efficiency and batch-to-batch consistency.

Scaling up production from laboratory-scale synthesis to industrial manufacturing also presents considerable technical challenges. Parameters that can be effectively controlled under small scale conditions may become difficult to reproduce during large scale production, potentially affecting nanoparticle quality, yield, and therapeutic performance. Consequently, the development of scalable and Good Manufacturing Practice (GMP) compliant production processes is essential for commercial implementation.

Intellectual property considerations further complicate commercialization efforts. Because many medicinal plants and extraction methods are based on traditional knowledge or naturally occurring resources, securing robust patent protection may be more challenging compared to proprietary synthetic platforms. In addition, biogenic nanoparticles must compete with established chemically synthesized nanomaterials that already benefit from optimized manufacturing processes, extensive characterization data, and clearer regulatory pathways [170].

Addressing these commercialization barriers will require coordinated efforts involving researchers, industry stakeholders, and regulatory authorities to establish standardized production methods, strengthen intellectual property strategies, and demonstrate the clinical and economic advantages of biogenic nanoparticle platforms.

### 8.9. Strengths and Limitations of Current Evidence

The current literature demonstrates several strengths of phytochemical corona mediated nanoparticles, including eco-friendly synthesis, multifunctional biological activity, intrinsic surface functionalization and potential applications against antimicrobial resistance and cancer.

Still, significant limitations remain in place. Most of the studies are confined to in vitro experiments with relatively few in vivo validations. The considerable heterogeneity in plant sources, extraction procedures, nanoparticle characterization methods and biological assays makes the reproduction and comparison of studies difficult. There are no standardized protocols for corona characterization, and long term safety and pharmacokinetics, immunogenicity and regulatory pathways are poorly understood. Many mechanistic studies also extrapolate phytochemical contributions without direct experimental verification of corona composition function relationships.

Future studies need to focus on standardized reporting frameworks, rigorous mechanistic validation, extensive toxicity studies, and clinically relevant translational models.

## 9. Future Directions and Emerging Opportunities

**Figure 2:**
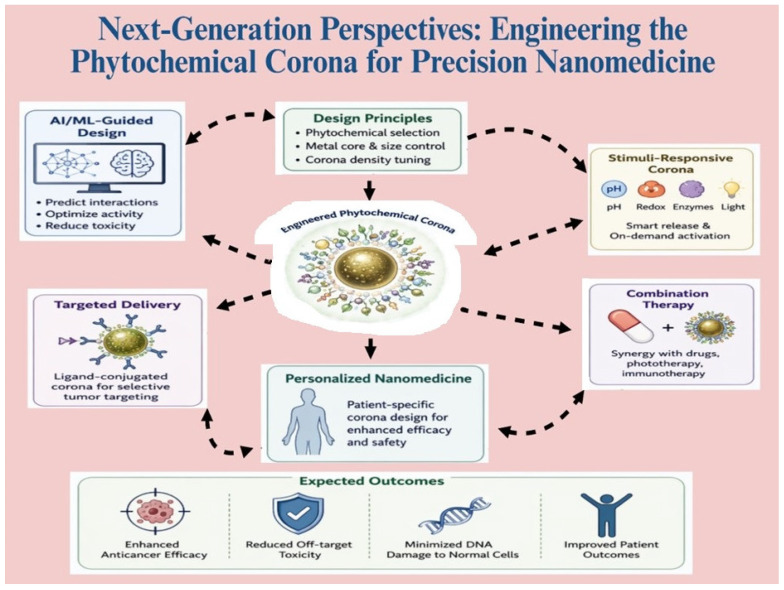
Schematic representation of integrated framework for engineering phytochemical corona functionalized biogenic nanoparticles for precision nanomedicine. Four interconnected design pillars—(i) AI/ML-guided design for predicting interactions, optimizing activity and reducing toxicity; (ii) rational design principles including phytochemical selection, metal core and size control, and corona density tuning; (iii) targeted delivery via ligand conjugated corona for selective tumor targeting; and (iv) personalized nanomedicine enabling patient specific corona design—converge toward four expected therapeutic outcomes: enhanced anticancer efficacy, reduced off target toxicity, minimized DNA damage to normal cells, and improved overall patient outcomes. This corona-focused engineering paradigm shifts the field from empirical synthesis to mechanism-driven, predictive design in biogenic nanomedicine. (Source: BioRender.com).

Figure 2 summarizes the main argument of this review, which is that the advancement of biogenic nanomedicine relies on the transition from empirical, trial-and-error synthesis to a rational, mechanism-guided design of the phytochemical corona. This holistic framework of AI/ML-enabled predictive optimization, rational phytochemical selection, targeted ligand decoration and patient specific customization provides a lucid roadmap for translating phytochemical corona nanoparticles into clinically viable precision therapeutics with enhanced efficacy and minimal off-target toxicity [105].

### 9.1. Phytosynthesis Using AI

Artificial intelligence and machine learning are increasingly making the prediction of nanoparticle synthesis feasible. Supervised learning models can predict particle size, surface charge and uniformity from the synthesis conditions. These models further link nanoparticle properties with biological activity [171].

### 9.2. Nanomedicine Precision

Precision nanomedicine tailors nanoparticle therapies for individual patients by using their genetic and protein profiles. For example, PUMA-activating coronas could be used to treat p53 mutant cancers, and AKT-inhibiting coronas could be used to treat PI3K-driven cancers [131]. Theranostic nanoparticles combine therapeutic and diagnostic imaging functions on one platform [172].

### 9.3. Engineering the Nano–Bio Interface

The frontier of nanoparticle design is the rational engineering of the nano–bio interface [173]. Corona engineering of phytochemical composition modulates protein adsorption for predictable behavior in living organisms. The computational approaches such as molecular dynamics simulations [174] are used to predict the conformation of protein corona before the synthesis of nanoparticles.

### 9.4. Integration with Omics Technologies

Multi-omics approaches assist researchers in understanding how nanoparticles interact with cells and tissues [175]. Genomics screens for genetic determinants of cellular responses to nanoparticles [2,176]. Transcriptomics identifies cellular pathways that are up- or down-regulated after exposure to NPs [176]. Proteomics identifies the proteins binding to the nanoparticle surface and defines the biological identity of the particle [177]. Metabolomics maps plant-derived metabolites forming the outer corona surrounding biogenic nanoparticles [178]. Scientists can identify the key molecules responsible for therapeutic effects by integrating all data types [179].

### 9.5. Study of Endangered Indian Plants

Biogenic nanoparticles production can be a largely untapped resource in India’s endangered plant species [180]. Unique metabolites in rare plants can be used to develop nanoparticle coatings with new biological properties [180]. It is a conservation ethic that these plants should be used in a way that does not threaten wild populations [180]. The plants are grown using sustainable methods, growing plant cells in culture and using synthetic biology to make the desired compounds [180]. Bioprospecting agreements ensure that local communities receive fair remuneration for any benefits derived from their traditional knowledge and genetic resources [181].

### 9.6. Computational Nanomedicine and Digital Twins

Digital twins are virtual copies of a patient that allow researchers to test nanoparticles on a computer rather than in a living body [182]. These in silico models can predict the distribution of nanoparticles in the body, the binding efficacy to their target and the side effects they may induce [183]. The most important factors influencing the biodistribution of nanoparticles in the body are their physicochemical characteristics such as surface charge, size and coating material [184]. Advanced computational tools such as machine learning and multi-view learning frameworks are used to predict nanoparticle behavior and pharmacokinetics. Digital twins can also enable virtual trials and patient-specific dosing, thereby minimizing the need for animal experiments and accelerating the development of precision nanomedicines [182].

## 10. Methods

**Literature Search Strategy:** A literature search was conducted using the PubMed, Scopus, Web of Science, Google Scholar, and Science Direct databases. Additional relevant articles were identified through citation tracking of key publications. Search strategies employed combinations of relevant keywords such as “phytochemicals”, “secondary metabolites”, “bioactive compounds”, “biomedical applications”, pharmacological applications”, “antioxidant”, “antimicrobial”, and “cytotoxicity”. The search encompassed publications available up to March 2026, with priority given to peer-reviewed journal articles. For key words, Boolean operators (AND/OR) were used to refine searches.
**Time Period:** The literature search primarily focused on studies published between January 2005 and March 2026 to capture both foundational protein corona concepts and recent advances in phytochemical corona-mediated nanomedicine. Earlier landmark publications were included where scientifically relevant.
**Inclusion Criteria:** Peer-reviewed articles, reviews, original research, English language, and studies related to plant-mediated nanoparticle synthesis and biological applications.
**Exclusion Criteria:** Conference abstracts, non-English publications, duplicated studies, studies lacking nanoparticle characterization, and articles unrelated to phytochemical corona or biogenic nanoparticles.

## 11. Conclusions

The emerging concept of the phytochemical corona provides a valuable framework for understanding the biological behavior of biogenic metal nanoparticles. Rather than functioning merely as metallic cores coated with passive stabilizing agents, these nanoparticles possess surface-associated phytochemicals that contribute to their physicochemical properties and interactions with biological systems. Increasing evidence suggests that the composition of the phytochemical corona may influence nanoparticle stability, cellular recognition, immune responses, biodistribution and therapeutic activity. Consequently, adopting a corona-centric perspective may facilitate a more mechanistic understanding of nano–bio interactions and support the development of rational design strategies for biogenic nanomedicines.

India’s extensive medicinal plant diversity offers a unique opportunity to investigate how variations in phytochemical composition influence corona architecture and biological function. Medicinal plants such as *Azadirachta indica*, *Withania somnifera*, and *Tinospora cordifolia* contain diverse classes of bioactive metabolites, including polyphenols, flavonoids, terpenoids, alkaloids, and withanolides, which may contribute to the functional properties of biogenic nanoparticles. Understanding the relationships between specific phytochemical constituents and therapeutic outcomes could support the development of nanoparticles with improved efficacy against complex diseases. However, direct evidence linking individual phytochemicals within the corona to specific biological effects remains limited and requires further investigation.

Biogenic nanoparticles have demonstrated promising antimicrobial and anticancer activities through multiple mechanisms of action, including membrane disruption, modulation of oxidative stress, interference with biofilm formation, regulation of inflammatory pathways, and induction of regulated cell death processes. Such multifunctional properties may provide advantages in addressing challenges associated with antimicrobial resistance and therapeutic resistance in cancer. Still, most current evidence is derived from in vitro studies and preclinical models, emphasizing the need for rigorous in vivo validation and carefully designed translational studies.

Despite these promising developments, several challenges continue to delay the clinical translation of phytochemical corona-mediated nanomedicines. These include limited reproducibility arising from variations in plant composition, a lack of standardized extraction and synthesis protocols, insufficient characterization of phytochemical coronas, difficulties associated with large-scale manufacturing, and the absence of well-defined regulatory frameworks specific to biogenic nanoparticles. Addressing these limitations will require the implementation of standardized analytical methodologies, high-throughput characterization platforms, and Good Manufacturing Practice (GMP)-compliant production strategies.

Future advances in analytical chemistry, systems biology, computational modeling, and artificial intelligence may provide new opportunities to improve our understanding of phytochemical corona formation and function. Integrating multi-omics approaches with predictive modeling could facilitate the identification of relationships between corona composition and biological outcomes, thereby supporting more rational nanoparticle design. However, the successful application of these emerging technologies will depend on the availability of robust experimental datasets and standardized characterization procedures.

Overall, phytochemical coronas represent an important and evolving aspect of biogenic nanoparticle research that has the potential to advance the field of precision nanomedicine. While substantial scientific and translational challenges remain, continued interdisciplinary efforts integrating photochemistry, nanotechnology, materials science, computational approaches, and biomedical research may enable the development of safer, more effective, and clinically relevant biogenic nanotherapeutics. Further mechanistic investigations and rigorous translational studies will be crucial to determine whether phytochemical corona engineering can ultimately contribute to the next generation of nanomedicine platforms.

Indian medicinal flora, such as *Azadirachta indica* and *Moringa oleifera* species, are a rich and renewable resource for the green synthesis of metal nanoparticles with controlled morphology and significant biological activity [185]. The data show that nanoparticle morphology is not random but reflects the phytochemical grammar of each plant. The dominance of spheres (~70% of entries) suggests that isotropic growth is energetically favored, whereas the anisotropic shapes (hexagons, triangles, rods) reveal selective facet binding by certain flavonoids and terpenoids. Ultrasmall (<20 nm) nanoparticles of *Withania somnifera* and *Gymnosporia montana* are promising in biomedical applications due to the enhanced cellular uptake [186]. The presence of marine plants such as *Cymodocea serrulata* suggests that aquatic media have special phytochemical profiles that can be used to synthesize nanoparticles with different properties for anticancer and antioxidant applications. Indian flora has demonstrated versatility for synthesis of multiple types of nanoparticles (Table 2). *Andrographis echioides* and *Piper longum* are some of the plants which have been successfully used for synthesis of silver as well as zinc oxide nanoparticles. Nanomaterials of *Garcinia indica* suggest that fruit-based extracts can also be effective reducing and capping agents, thereby increasing the scope of plant tissues available for phytofabrication. In summary, the data confirm that the biogenic nanoparticle morphology and size are non-random and are reflective of the underlying ‘phytochemical grammar’ of each plant, which has direct implications for the tailoring of nanoparticle properties for specific therapeutic applications [185]. These findings add to the promise of Indian flora as a source of natural nanotechnology, but also highlight a pressing need for methodological standardization and rigorous characterization throughout independent research.

## Figures and Tables

**Table 1:** Methods for characterization of secondary metabolites from phytochemicals.

Technique	Purpose
LC-MS/MS	Metabolite identification
GC-MS	Volatile compounds
FTIR	Functional groups
Raman spectroscopy	Molecular fingerprints
NMR	Structural elucidation
XPS	Surface chemistry
HPLC	Quantification
MALDI-TOF MS	Corona profiling
Zeta potential	Surface charge
TGA	Corona mass estimation

**Table 2:** Size and morphology of biogenic nanoparticles from Indian flora.

Sr. No.	Plant Source	Nanoparticle Type	Size and Shape	Reference
1	*Achyranthes aspera*	Gold	20–30 nm, Spherical/hexagonal	[187]
2	*Padina tetrastromatica*	Gold	8–10 nm, Spherical	[188]
3	*Bauhinia tomentosa*	Gold	16–31 nm, Spherical	[189]
4	*Melia azedarach*	Silver	76 nm, Cubical/spherical	[190]
5	*Moringa oleifera*	Silver	38–40 nm, Spherical	[191]
6	*Podophyllum hexandrum*	Silver	14 nm, Spherical	[192]
7	*Cymodocea serrulata*	Silver	29.28 nm, Spherical	[193]
8	*Heliotropium indicum*	Silver	80–120 nm, Spherical	[194]
9	*Syzygium cumini*	Silver	40 nm, Spherical/hexagonal	[195]
10	*Calotropis gigantea*	Silver	5–30 nm, Spherical	[196]
11	*Acorus calamus*	Silver	36 nm, Spherical	[197]
12	*Commelina nudiflora*	Gold	24–80 nm, Spherical/triangular	[198]
13	*Andrographis echioides*	Silver	68 nm, Pentagonal/hexagonal	[199]
14	*Alternanthera sessilis*	Silver	10–30 nm, Spherical	[200]
15	*Vitex negundo*	Silver	22 nm, Spherical	[201]
16	*Coriandrum sativum*	Silver	20–80 nm, Spherical	[202]
17	*Acorus calamus*	Silver	59 nm, Spherical/cuboidal	[197]
18	*Rosa indica*	Silver	23–60 nm, Spherical	[203]
19	*Cucurbita maxima*	Silver	76 nm, Spherical/cuboidal	[204]
20	*Syzygium aromaticum*	Silver	5–20 nm, Spherical	[205]
21	*Gossypium hirsutum*	Silver	13–40 nm, Spherical	[206]
22	*Scoparia dulcis*	Silver	15–25 nm, Spherical	[207]
23	*Origanum vulgare*	Silver	136 nm, Spherical	[208]
24	*Erythrina indica*	Silver	20–218 nm, Spherical	[209]
25	*Rosa damascena*	Silver	20–80 nm, Spherical	[210]
26	*Piper longum*	Silver	46 nm, Spherical	[211]
27	*Suaeda monoica*	Silver	31 nm, Spherical	[212]
28	*Lasiosiphon eriocephalus*	Gold	20–60 nm, Hexagonal	[213]
29	*Argemone mexicana*	Gold	20–40 nm, Hexagonal	[214]
30	*Nothapodytes foetida*	Gold	20–200 nm, Hexagonal/polygonal	[215]
31	*Acorus calamus*	Gold	10–100 nm, Spherical	[216]
32	*Gymnosporia montana*	Gold	2–20 nm, Irregular/spherical	[217]
33	*Amaranthus spinosus*	Gold	10–20 nm, Spherical	[218]
34	*Terminalia arjuna*	Gold	20–50 nm, Spherical	[219]
35	*Azima tetracantha*	Gold	80 nm, Spherical	[220]
36	*Desmodium gangeticum*	Gold	12–20 nm, Triangular/spherical	[221]
37	*Thespesia lampas*	Gold	12–45 nm, Spherical	[222]
38	*Withania somnifera*	Gold	3–10 nm, Hexagonal	[223]
39	*Garcinia indica*	Gold	8–11 nm, Spherical/triangular	[224]
40	*Elephantopus scaber*	Gold	20–40 nm, Spherical	[225]
41	*Artemisia vulgaris*	Gold	50–100 nm, Spherical/hexagonal	[226]
42	*Clitoria ternatea*	Gold	100 nm, Rod-shaped	[227]
43	*Murraya koenigii*	Gold	20–40 nm, Spherical	[228]
44	*Artocarpus hirsutus*	Gold	5–40 nm, Spherical	[229]
45	*Terminalia arjuna*	Gold	20–25 nm, Spherical	[219]
46	*Memecylon umbellatum*	Gold	15–25 nm, Spherical/hexagonal	[230]
47	*Coreopsis lanceolata*	Gold	20–30 nm, Spherical	[231]
48	*Cassia auriculata*	Gold	15–25 nm, Spherical/hexagonal	[232]
49	*Nyctanthes arbor-tristis*	Gold	15–25 nm, Spherical	[233]
50	*Guazuma ulmifolia*	Gold	20–25 nm, Spherical	[234]
51	*Musa paradisiaca*	Gold	50 nm, Spherical	[235]
52	*Terminalia arjuna*	Gold	60 nm, Triangular/hexagonal	[236]
53	*Lantana camara*	Gold	150–300 nm, Triangular	[237]
54	*Citrus limon*	Gold	32–56 nm, Spherical/triangular	[238]
55	*Abelmoschus esculentus*	Gold	45–75 nm, Spherical	[239]
56	*Theobroma cacao*	Gold	150–200 nm, Spherical	[240]
57	*Momordica cochinchinensis*	Gold	10–80 nm, Spherical/oval/triangular	[241]

## Data Availability

No new data were created or analyzed in this study. Data sharing is not applicable to this article.

## Abbreviations

AMR	Antimicrobial Resistance.
PUMA	p53 upregulated mediator of Apoptosis.
P53	protein 53.
PEG	Polyethelyene glycol.
ROS	Reactive Oxygen Species.
NF-κB	Nuclear Factor kappa-light-chain-enhancer of activated B cells.
Nrf2	Nuclear factor erythroid 2-related factor 2.
AKT	Protein Kinase B (PKB).
TNF-α	Tumor Necrosis Factor-alpha.
Bax	Bcl-2-associated X protein.
FOXO	Forkhead box O.
JNK	c-Jun N-terminal kinase.
HER2	Human Epidermal Growth Factor Receptor 2.
MAPK	Mitogen-Activated Protein Kinase.
EGFR	Epidermal Growth Factor Receptor.
